# Glucose 6 phosphate dehydrogenase deficiency and hemoglobinopathy in South Western Region Nepal: a boon or burden

**DOI:** 10.1186/s13104-019-4762-6

**Published:** 2019-11-08

**Authors:** Narayan Gautam, Bhagwati Gaire, Trishna Manandhar, Bishnu P. Marasini, Niranjan Parajuli, Sunil P. Lekhak, Monica Nepal

**Affiliations:** 1 Department of Biochemistry, Universal College of Medical Sciences, Bhairahawa, Nepal; 2 Department of Biotechnology, National College, Kathmandu, Nepal; 3 Central Department of Chemistry, Tribhuvan University, Kathmandu, Nepal; 4 Decode Genomics and Research Center, Kathmandu, Nepal; 5 Department of Pharmacological and Bio-Molecular Sciences, Universita Degli Studi Di Milano, Milan, Italy

**Keywords:** G6PD, Hemoglobinopathy, SCA, SCT, Tharu community, β-TT

## Abstract

**Objectives:**

The study was carried out to optimize the phenotypic method to characterize the sickle cell trait (SCT), sickle cell anemia (SCA), and β-thalassemia (β-TT) suspected sample from tharu community of South Western province-5, Nepal. SCT and SCA were further evaluated by genotypic method employing amplification refractory mutation system (ARMS PCR). Moreover, Glucose 6 phosphate dehydrogenase (G6PD) was estimated in those hemoglobinopathy to observe its prevalence. The accurate and reliable method can play an important role in reduction of morbidity and mortality rate.

**Results:**

The 100 suspected cases were subjected to phenotypic method adopting cellulose acetate electrophoresis and genotypic method using ARMS PCR which portraits (5%) SCA positive test showing HBS/HBS, (38%) SCT positive trait HBA/HBS and (36%) cases normal HBA/HBA. β-TT (21%) cases were confirmed by electropherogram. G6PD deficiency was observed in (40%) of SCA, (18.4%) of SCT, (4.8%) of β-TT and (2.8%) in normal cases. Increased G6PD were developed only in SCT (5.3%) and β-TT (4.8%). The study highlighted sickle cell disorder (SCD) and β-TT as the most common hemoglobinopathy coexisting with G6PD deficiency. Though hemoglobinopathy sometime could be protective in malaria but G6PD deficiency can cause massive hemolysis which may exacerbate the condition.

## Introduction

Congenital causes of hemolysis includes enzymopathy like glucose 6 phosphate dehydrogenase (G6PD) deficiency, several hemoglobinopathy like sickle cell anemia (SCA) and thalassemias. G6PD is vital enzyme responsible for tackling various oxidative stresses that red blood cells (RBCs) constantly face, preventing hemolysis [1].

Sickle cell disease (SCD) is one of the most important disorders caused by variant of β globin protein of hemoglobin called hemoglobin S (HBS). Individuals with homozygous HBS express SCA. Heterozygosity with one copy normal β globin gene of HBA is not associated with any clinical defect. Individual SCT have 50 percent chance of passing gene to offspring and have few complications than SCA. The clinical consequences can be hematological complications, vasoocclusion and organ dysfunction which can undergo pain episode, also known as a sickle cell crisis which occurs when tissue become damage [2].

The estimated SCD is found to be 30,000 and most of tharus have sickle cell throughout the country with heavy prevalence in mid and far western regions of Nepal [3]. Prevalence of thalassemia in Nepal is particularly high among tharus with gene frequency of 0.8 [4]. Hemoglobinopathy and G6PD deficiency have been documented over various regions of world including Nepal, particularly in tharu community of southwestern terai region [5, 6]. The reason for such high prevalence in these areas traces back to strong selective pressure that malaria exerts on shaping the human genome for at least 6000 years [7].

Though exact data regarding the spectrum of hemoglobinopathy in Nepal is not known, it seems to be increasing. If disease continues to pass vertically, it may take the form of epidemic. This study has provided necessary framework for generation of data from these areas to include in national health management system regarding phenotypic attributes of hemoglobin disorders associated with G6PD status among tharus. The aim of the study was molecular characterization for accurate diagnosis of SCD and its association with G6PD status. Moreover, β-TT phenotypes and its association with G6PD status have been shown.

## Main text

### Methods

This community based cross sectional study was carried out in total of 100 suspected cases of South Western Region, province-5, Nepal. Districts with dominance of tharu ethnicity were chosen viz a viz Nawalparasi, Rupandehi, Kapilvastu, Dang and Bheri. The study was conducted from May to December, 2018. The sample was collected in EDTA vial and hemolysate was kept at − 20 ℃ prior to application of hemoglobin electrophoresis in the Department of Biochemistry, UCMS, Bhairahawa. The sample with or without clear bands phenotypically in cellulose acetate electropherogram for SCD was proceed further for molecular test to screen clearly normal trait and disease condition by applying ARMS PCR in the Department of Biotechnology, National College, Kathmandu.

### Cellulose acetate electrophoresis

This technique is based on the principal of electrophoresis that mainly separates HBA, HBS, HBA_2_ and other forms of hemoglobin variants used in screening SCD and thalassemia [8]. Cellulose acetate electrophoresis was performed at alkaline pH (8.6) on the prepared hemolysate from the blood sample to assess the spectrum of hemoglobinopathy.

The hemolysate preparation was the initial step of cellulose acetate electrophoresis. It starts up with fresh anticoagulant blood (2 ml). After the centrifugation at 2000 to 5000 rpm two layers were obtained, upper layer was plasma which was discarded and lower layer was the RBC. Double volume of normal saline (0.85%) was added to the pellet, this process was repeated for three times to remove plasma. Again centrifugation was performed at 3000 to 5000 rpm for 3 min and plasma was discarded. Three to five drops of distilled water was added to lyse RBC. Two millilitre chloroform was added and mixed; centrifuge was performed at 5000 rpm for half an hour. The top most layers obtained after centrifugation was hemolysate layer, collected in fresh tube and stored at − 20 °C for further use.

The cellulose acetate tank was set up with buffer. The cellulose acetate membrane was adjusted to the buffer tank filled with (1×) Tris EDTA Borate buffer (TEB) pH 8.6. It was adjusted with the help of filter paper on both ends by making wicks. Ten microliter sample was loaded on different sample tray and was noted carefully for the sample position. Then, with the help of applicator the loaded samples were touched and the same applicator sample was transferred on the cellulose acetate membrane on the buffer tank. Immediately electrophoresis was carried out with 150 V and 2 amp current for 30 min. After that staining was done with 0.1% ponceau S for a minute. The destaining was performed with 2% acetic acid until the clear band was observed and finally analyzed for different bands.

### ARMS PCR

It is the method for detecting any single base pair mutation or deletion. This method was applied to detect sickle cell allele by using two ARMS primers as OF-OR primer position 311, IF-OR primer position 146 and OF-IR primer position 203 [9].

The genomic DNA obtained was visualized in 0.4% agarose gel along with ladder. After, conformation of genomic DNA, ARMS PCR was carried out. As per optimized PCR condition, the reaction volume with sickle forward and reverse primer (10 μM) of total 25 μl reaction was carried out in a single tube for 1.5 h. Thereafter, tube was taken out and electrophoresis of PCR product was performed with 2% agarose gel. Finally amplification of PCR product was analyzed.

**Table:** Primers sequences and its band positions

Primers	Sequence of primer (band position)	Number of band position	Types of mutations
OF-OR	CTTAGACCTCACCCTGTGGAGACATGC CCA GTT TCTATTGGT (311)	311 and 146	HBA/HBA (normal)
IF-OR	TGGTGCATCTGACTCCAGA ACA TGC CCA GTT TCTATTGGT (146)	311,203 and 146	HBA/HBS (heterozygous)
OF-IR	CTTAGACCTCACCCTGTGGAG AGT AAC GGC AGA CTT CTGCA (203)	311 and 203	HBS/HBS (homozygous mutant)

### G6PD assay

G6PD in RBCs is released by lysing agent present in the reagent. The G6PD released catalyzes the Glucose-6-phosphate with reduction of NADP to NADPH. The rate of reduction of NADP to NADPH is measured as an increased in absorbance at 340 nm produced in the reaction catalyzed by the enzyme which is proportional to the G6PDH activity in the sample [10]. Coral G6PD assay kit (Clinical System, Bambolim complex, Goa, India) was used and the procedure provided in the manual with the kit was followed. The absorbance was taken 2 min after reaction mixture was added with blood and final value was calculated by multiplying absorbance (∆A) with factor 4778 divided by hemoglobin concentration of the patients which was determined by CelltacEs MEK-7300K, 5-part hematology analyzer. The normal value of the G6PD activity at 37 °C is 6.4–18.7 IU/g Hb.

Ethical approval for the research (UCMS/IRC/73/18) was taken from Institutional Review Committee (IRC), UCMS, Bhairahawa, Nepal. Consent was taken from the participants and concerned guardians prior to the study. Data were analyzed by Statistical Package for Social Service (SPSS) version 22, Inc. Chicago, IL. Data were expressed in terms of frequency (%) for categorical data and Chi square test was applied to analyze the level of significance at *p* value < 0.05. Odd ratio (OR) was calculated in 95% confidence interval (CI) to assess the risk factor.

## Results

In our study, 100 suspected hemoglobinopathy cases were examined by history, sign of anemia and jaundice in tharu community from high incidence area of south western regions province-5 including Nawalparasi, Rupandehi, Kapilvastu, Dang and Bheri.

**Fig. 1:**
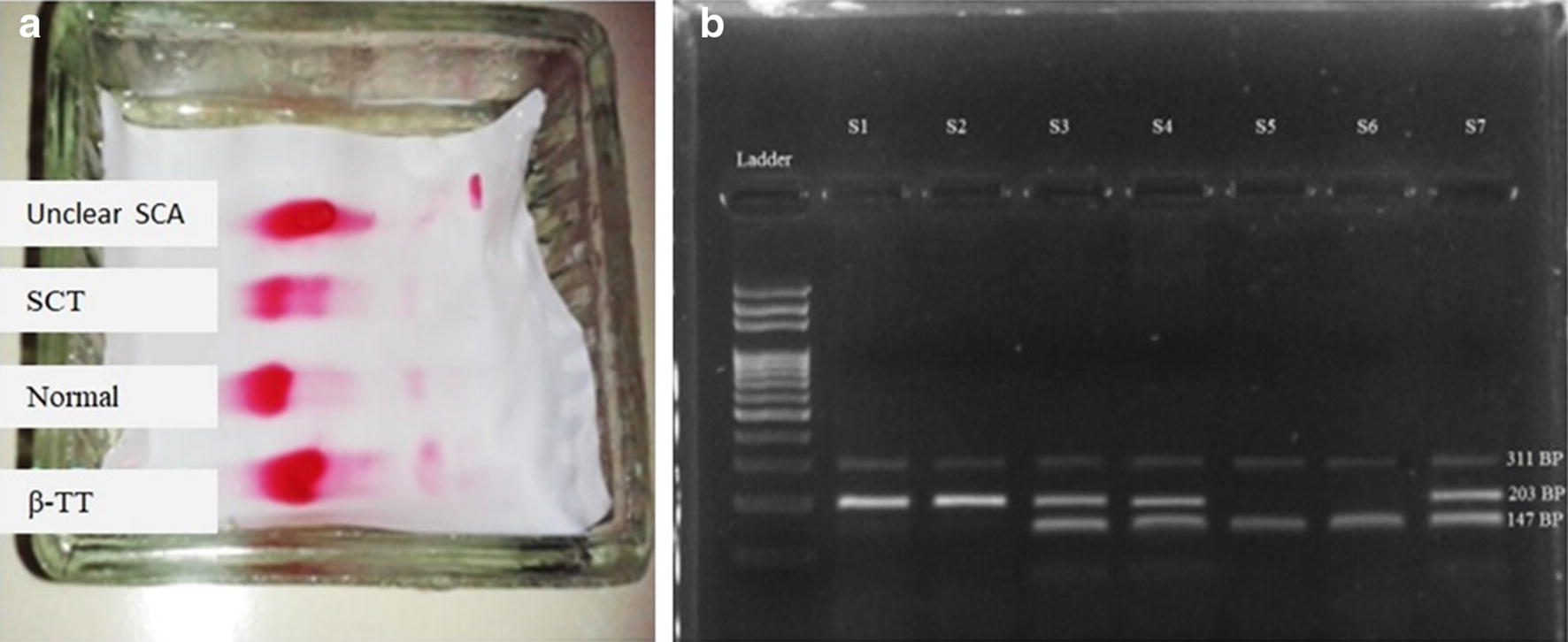
**a** Cellulose acetate electrophoresis at pH 8.6. **b** ARMS PCR amplification of genomic DNA sample showing sickle cell mutation, heterozygonus mutation and normal β globin gene

Electropherogram was visualized after destaining with 2% acetic acid solution in cellulose acetate which was initially run at 150 V and 2 amp current for 0.5 h. In Fig. 1a; lane 1 represents unclear SCA (HBS/HBS) bands, lane 2 represents positive SCT (HBA/HBS) bands, lane 3 Normal (HBA/HBA) bands, and lane 4 represents β-TT with Increased HBA_2_ band. Bands which were not explicit and others that were clear for SCD; both have been further processed by ARMS PCR to get accurate result.

DNA was amplified by using conventional PCR method with the mixture of Thermus aquaticus (Taq) DNA polymerase, master mix, designed primer, nuclease free water and DNA volume. The amplified products were run on 2% agarose gel at 150 V for 20 min along with 100 bp ladder. Thus, all result obtained were at same position as per primer design. In Fig. 1b; lane 1 represents 100 bp ladder starting from 100 bp (least) and lane 2 (S1) and 3 (S2) represent positive sample SCA denoting (HBS/HBS) band position at 311 bp and 203 bp. Lane 4 (S3), 5 (S4), 8 (S7) represents heterozygous condition SCT denoting (HBA/HBS) band position at 311, 203 and 147 bp. Lane 6 (S5) and 7 (S6) represent normal β globin gene denoted by (HBA/HBA) band position at 311 and 147 bp.

As presented in Table 1, G6PD groups were categorized into normal, deficient and increased. Among the 100 cases, 86% had normal G6PD levels, 11% had G6PD deficiency and 3% had increased G6PD levels. The maximum age group with G6PD deficiency was observed in 45–65 years (17.6%) followed by ≥ 60 years (15.4%) and children 12–14 years (15%). Of the deficient group, most of the cases were male (81.8%). G6PD group was analyzed according to regional distribution with higher in Dang (36.4%) followed by Bheri (27.3%).

**Table 1:** Comparison of frequency (%) distribution of G6PD group with study variables

	G6PD (IU/g Hb) group			Total	p-value
	Normal 6.4–18.7	Deficiency < 6.4	Increased > 18.7		
Age group (years)					
12–14	17 (85%)	3 (15%)	0 (0%)	20	0.745
15–29	26 (86.7%)	2 (6.7%)	2 (6.7%)	30	
30–44	18 (90%)	1 (5%)	1 (5%)	20	
45–60	14 (82.4%)	3 (17.6%)	0 (0%)	17	
≥ 60	11 (84.6%)	2 (15.4%)	0 (0%)	13	
Gender	Normal	Deficiency	Increased		
Male	39 (45.3%)	9 (81.8%)	1 (33.5%)	49	0.053
Female	47 (54.7%)	2 (18.2%)	2 (66.7%)	51	
Region (Province no. 5)	Normal	Deficiency	Increased		
Nawalparasi	18 (20.9%)	2 (18.2%)	0 (0%)	20	0.406
Rupandehi	17 (19.8%)	1 (9.1%)	0 (0%)	18	
Kapilvastu	19 (22.1%)	1 (9.1%)	0 (0%)	20	
Dang	16 (18.6%)	4 (36.4%)	1 (33.3%)	21	
Bheri	16 (18.6%)	3 (27.3%)	2 (66.7%)	21	
Diagnosis	Normal	Deficiency	Increased		
Normal	35 (97.2%)	1 (2.8%)	0 (0%)	36	0.063
SCT	29 (76.3%)	7 (18.4%)	2 (5.3%)	38	
SCA	3 (60%)	2 (40%)	0 (0%)	5	
β-TT	19 (90.5%)	1 (4.8%)	1 (4.8%)	21	
Anemia (WHO criteria)	Normal	Deficiency	Increased		
Normal [> 13 (M), > 12 (F) g/dl]	7 (100%)	0 (0%)	0 (0%)	7	0.574
Mild [11–12.9 (M) 11–11.9 (F) g/dl]	22 (84.6%)	4 (15.4%)	0 (0%)	26	
Moderate [8–10.9 (M/F) g/dl]	42 (82.4%)	6 (11.8%)	3 (5.9%)	51	
Severe [< 8 (M/F) g/dl)	15 (93.8%)	1 (6.3%)	0 (0%)	16	

When explored for hemoglobinopathy through cellulose acetate electrophoresis, 38% had SCT, 5% had SCA, 21% had β-TT and 36% had no hemoglobinopathy. The problem with band clarity in cellulose acetate, all SCD and normal results were run ARMS PCR to reconfirm SCD diagnosis. G6PD deficiency was more prevalent in the SCA group (40%) followed by SCT (18.2%), β-TT (4.8%) and Normal (2.1%). G6PD deficiency was observed in 15.4% of mild anemia cases, 11.8% of moderate anemia cases and 6.3% of severe anemia cases.

As represented in Table 2 male had higher risk of having G6PD deficiency as compared to females (OR 5.01, p = 0.035). Odds ratio was also high (OR 6.1, p = 0.078) for hemoglobinopathy group, with SCD and β-TT as compared to their normal peer.

**Table 2:** Risk factors of G6PD deficiency with study variables

Dependent variable	Non-dependent variable	OR	95% CI	p-value
G6PD deficiency	Sex = Male	5.01	1.12–26.98	0.035
	Age = Adult ≥ 15 years	1.58	0.38–6.62	0.52
	Region = Bheri and Dang	2.7	0.73–9.90	0.134
	Diagnosis = SCT, SCA and β-TT	6.10	0.80–53.9	0.078

## Discussion

In Nepal SCD affects mostly the tharus residing in western part Kailali, Kanchanpur, Dang, Bardiya [11]. So, diagnostic tool used for detection of SCD should be precise and accurate.

In cellulose acetate method, the analysis of the band is quite difficult as compared to other molecular tools. The separated bands of HBA and HBS appeared closer to each other so its prominent detection and analysis sometime become difficult. In present study, electropherogram is capable of differentiating normal samples from SCD and β-TT but the variant SCA and SCT weren’t prominent. Hence, ARMS PCR was optimized to detect single point mutation on all those samples with the specific primer alignment can be accomplished accurately. The cellulose acetate electrophoresis is standard phenotype detection method but clear segregated bands couldn’t be obtained regards to hemoglobin variants as a wide range of band occurred nearby same position. In congruence to the current study, Akkani et al. concluded that molecular method was reliable tool for characterization of SCD [12].

The qualitative tests most commonly used to check for G6PD deficiency in clinical settings are adequate for identifying males with G6PD deficiency, thus informing appropriate treatment options. However, these tests do not accurately define G6PD activity in females, potentially exposing women with intermediate G6PD activity to the risk of severe anemia, hemolysis and other health impacts [13]. The present study highlighted the highest prevalence of G6PD deficiency in Dang and Bheri [11], [14]. The studies conducted in Afghanistan, Bangladesh, Bhutan, India, Nepal, and Pakistan found that the G6PD deficiency prevalence ranges from 3.8 to 15%, with endemic areas of Nepal [15]. Overall 11% prevalence in G6PD deficiency was observed.

G6PD deficiency was observed in 40% SCA, 18.4% SCT and 4.8% β-TT. Increased G6PD level was developed only in 5.3% SCT and 4.8% β-TT. In one case (2.8%) G6PD deficiency was also observed in normal cases. The study done by Bienzle et al. have shown 16% G6PD deficiency and 84% normal G6PD. The clinical course of SCD, including the degree of anemia was not milder in G6PD deficiency than in G6PD normal patients but could not be proved to be significantly more severe [16]. Most of the G6PD have neither anemia nor evidence of increased RBC destruction, nor an alteration in blood morphology, although a modest shortening of RBC survival can be demonstrated by isotopic techniques. However, episodes of acute hemolysis with hemolytic anemia may be triggered by medications, certain foods, and acute illnesses, especially infection [17]. In present study, the G6PD deficiency was higher in mild anemia followed by moderate and than severe anemia which might be due to lack of the history taking that triggers acute hemolytic attack in G6PD deficiency cases.

Direct testing of the enzymatic activity of G6PD on a freshly collected blood sample is the most widely used diagnostic method [18]. Rashid et al. have shown the overall prevalence of G6PD deficiency was 12.0% for children with ≤ 60% normal activity (≤ 3.0 U/g Hb). However, the prevalence of severe G6PD deficiency (≤ 10%; ≤ 0.5 U/g Hb) was 2.3% [19]. In addition, the G6PD deficiency prevalence rate is lower than the rates in malaria endemic areas in sub-Saharan Africa, where rates as high as 30% have been reported [20].

This community based study will help people residing those area to understand problems and enable them to prevent constellation of consequences of hemoglobin disorders existing with G6PD deficiency which are inherited single gene defects. This study can provide data to help in conducting awareness program in those affected communities.

## Conclusion

The study highlighted SCT and β-TT as the most common disorder in tharu community with G6PD deficiency co-existing with those conditions. Though hemoglobinopathy sometime could be protective in malaria associated morbidity but G6PD deficiency can be associated with hemolysis which may exacerbate the condition.

## Limitations

In this study we have tried to introduce molecular techniques ARMS PCR for SCD but not for β-TT and G6PD gene detection which are needed to be done with specific primers. Exact burden of hemoglobinopathy based on ethnic and geographic distribution can be achieved by multi-centric community based studies.

## Data Availability

The data set used and analyzed in this study is available from the corresponding author upon reasonable request.

## Abbreviations

β-TT: β thalassemia
G6PD: glucose 6 phosphate dehydrogenase
SCT: sickle cell trait
SCA: sickle cell anemia
SCD: sickle cell diseases
ARMS PCR: amplication refractory mutation system polymerase chain reaction
HBS: sickle cell hemoglobin
HBA: adult hemoglobin
UCMS: Universal College of Medical Sciences
